# Ectopic thymic tissue in subglottis of children: evaluation and management^⋆^

**DOI:** 10.1016/j.bjorl.2021.10.001

**Published:** 2021-11-05

**Authors:** Yihang Lin, Junyang Li, Lianyan Xue, Peixuan Sun, Qiao He, Youjin Li

**Affiliations:** a Shanghai Jiaotong University, School of Medicine, Shanghai Children’s Medical Center, Department of Otolaryngology—Head & Neck Surgery, Shanghai, China; b Shanghai Jiaotong University, School of Medicine, Shanghai Children’s Medical Center, Department of Radiology, Shanghai, China; c Shanghai Jiaotong University, School of Medicine, Shanghai Children’s Medical Center, Department of Diagnostic Imaging Center, Shanghai, China; d Shanghai Jiaotong University, School of Medicine, Shanghai Children’s Medical Center, Department of Pathology, Shanghai, China

**Keywords:** Congenital anomaly, Ectopic thymus, Subglottis

## Abstract

**Objectives:**

Ectopic thymic tissue in the subglottis is an extremely rare disease that causes airway obstruction. Few cases reported were accurately diagnosed before surgery.

**Methods:**

A case of a 2-year-old boy with airway obstruction caused by a left subglottic mass was reported. The presentation of radiological imaging, direct laryngoscopy and bronchoscopy, pathology, and surgical management were reviewed. An extensive search in PubMed, EMBASE, Web of Science, Google Scholar, and EBSCO of English literature was performed without a limit of time.

**Results:**

Besides our case, only six cases were reported since 1987. The definitive diagnosis on these patients were made with the findings of pathology, of which, five were ectopic thymus and two were ectopic thymic cysts. Our case was the only one with a correct suspicion preoperatively. Four cases underwent open surgical resection, and two cases underwent microlaryngeal surgery, while one deceased after emergency tracheostomy. No recurrences were found by six patients during the follow-up after successful treatments.

**Conclusion:**

Ectopic thymus is a rare condition, infrequently considered in the differential diagnosis of subglottic masses. Modified laryngofissure may be an effective approach to removing the subglottic ectopic thymus and reconstructing the intact subglottic mucosa.

## Highlights

• Subglottic ectopic thymic tissue is rare and unexpected cause of stridor in children.
• The knowledge of the typical ultrasonographic features can provide a hint to make a definitive diagnosis before surgery.
• Modified laryngofissure may be an effective approach to removing the subglottic ectopic thymus and reconstructing the intact subglottic mucosa.

## Introduction

Ectopic thymic tissue in the subglottis is an extremely rare disease, which causes airway obstruction. According to the definition, aberrant cervical thymus denotes the presence of thymic tissue in any area (such as the pharynx, trachea, neck, mediastinum, or esophagus) outside its normal descent path.^1^ Until now, only six cases of subglottic ectopic thymus have been reported in the English literature. Unlike the previous cases, here we reported a case of a child, in whom the ectopic thymus was suspected before the surgery. In this study, we highlighted the diagnosis and treatment of this rare disease through a report of a successfully treated case and briefly reviewed the English literature on the subglottic ectopic thymus.

## Methods

We undertook a review of literature published in English language with focus on ectopic thymus of the subglottic area. Databases searched in this study included PubMed, Embase, Web of Science, Google Scholar, and EBSCO. The literatures search was not limited by time. Search terms included ectopic thymus, subglottic stenosis, subglottic mass, and laryngeal and subglottic mass. Reference lists of the found articles were scanned for other case reports.

## Results

A 2-year-old boy with recurrent stridor and multiple episodes of acute laryngitis and progressive symptoms of upper airway obstruction was admitted to our department in June 2020. General physical examination showed normal lung and heart function and no signs of respiratory tract infection. No palpable neck mass was found. He had no swallowing difficulty or hoarse cry. Normal vocal cord motion was identified by flexible fiberoptic laryngoscopy.

**Figure 1:**
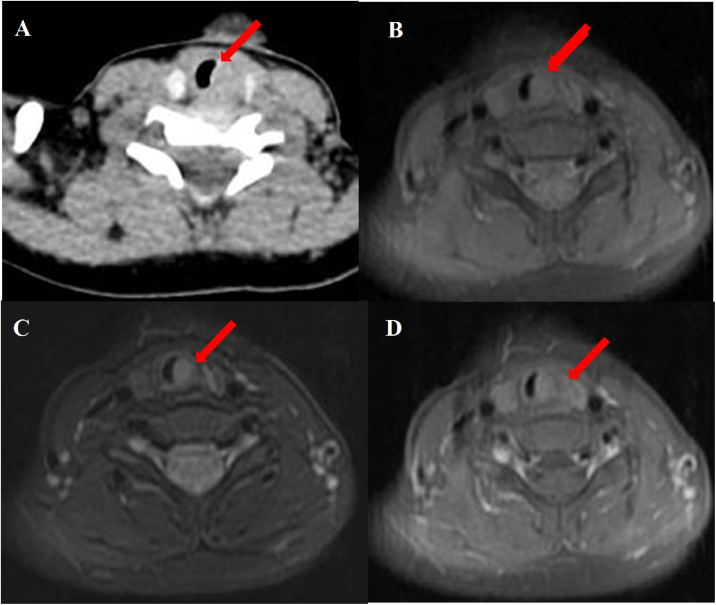
Subglottic masses in the 2-year-old boy presenting with stridor. Computed Tomography (CT) and Magnetic Resonance Imaging (MRI) scan of the neck showed (A) an abnormal soft-tissue density shadow on the medial side of bilateral thyroid upper pole with corresponding slightly narrowed airway (arrow). The thymus shadow was larger and seemed to extend to the neck. Transverse view of T1-weighted and Iterative Decomposition of water and fat with Echo Asymmetric and Least-squares estimation (IDEAL) T2-weighted MRI showed (B) isointense T1 and (C) hyperintense T2 masses in left of the trachea at the level of the thyroid gland and at the edge of the left and right lobe of the thyroid gland. (D) The masses enhanced slightly in contrast-T1-weighted transverse view. The trachea (arrow) was locally narrowed at the level of the thyroid gland.

**Figure 2:**
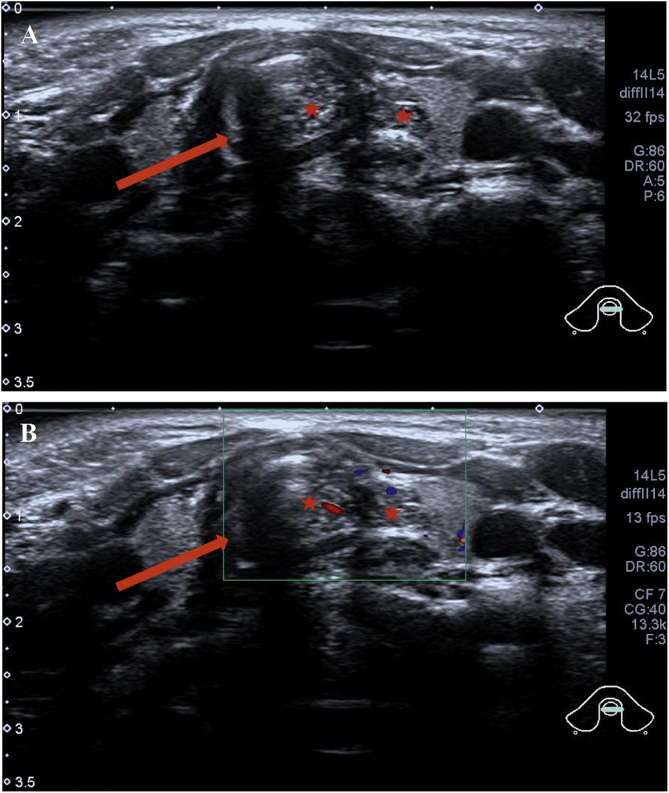
Intra-tracheal extension of the thymus in the 2-year-old boy presenting with stridor. Ultrasound showed (A) the tracheal ring (the dark area marked by an arrow) and thymus tissue (stars). Ectopic thymus was seen invading the trachea and presenting as heterogeneous hypoechoic masses of lymph nodes. (B) Color Doppler Flow Imaging (CDFI) blood flow chart; tracheal ring (arrow); ectopic thymus tissue (stars).

Abnormal signal was found at the thyroid level on neck Magnetic Resonance Imaging (MRI) scan (Fig. 1B–D); the abnormal signal measuring 7.8 × 5.7 × 12.6 mm was located on the left side to the trachea surrounding the thyroid gland. T1-weighted isointense and Iterative Decomposition of water and fat with Echo Asymmetric and Least-squares estimation (IDEAL) T2-weighted hyperintense signals were observed, slightly enhanced in contrast-T1-weighted view. The lesion causing tracheal compression was likely partially connected to the thymus in the root of neck. Computed Tomography (CT) scan demonstrated the abnormal soft-tissue mass at the superior pole of both thyroid lobes with no clear border and no obvious enhancement, accompanied by subglottic obstruction. CT value of the mass was 76HU, followed by 80HU after contrast administration (Fig. 1A). Ultrasonographic appearance through the neck showed a hypoechoic lesion, characterized by multiple internal echogenic foci giving a “starry sky” appearance and ill-defined borders in the posterior aspect of the superior part of the left thyroid lobe (Fig. 2), which was similar to the thymus in the anterior mediastinum, suggesting the possibility of ectopic thymic tissue.

**Figure 3:**
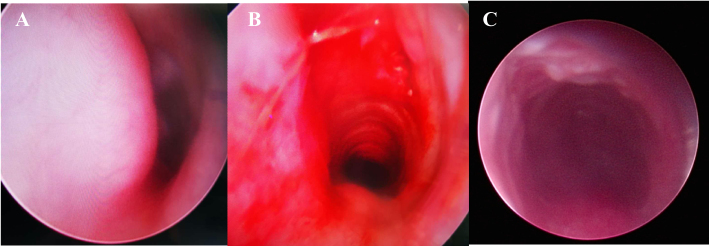
Direct Laryngoscopy and Bronchoscopy (DLB) showed (A) a subglottic semicircular mass with smooth mucosa, obstructed 70% laryngeal cavity (pre-operation). (B) Subglottic obstruction was significantly relieved (immediate post-operation). (C) Laryngeal obstruction was significantly relieved (a week after surgery).

Direct Laryngoscopy and Bronchoscopy (DLB) examination under general anesthesia was performed, revealing a subglottic semicircular mass with smooth surface mucosa and obstruction of 70% of the subglottic cavity (Fig. 3). Modified laryngofissure was performed to remove the submucosal soft tissue and retain the intact subglottic mucosa. The patient remained intubated postoperatively with an oral Endotracheal Tube (ETT). After surgery, the patient was further monitored in Pediatric Intensive Care Unit (PICU) and received analgesia and sedation as well as symptomatic support treatment.

**Figure 4:**
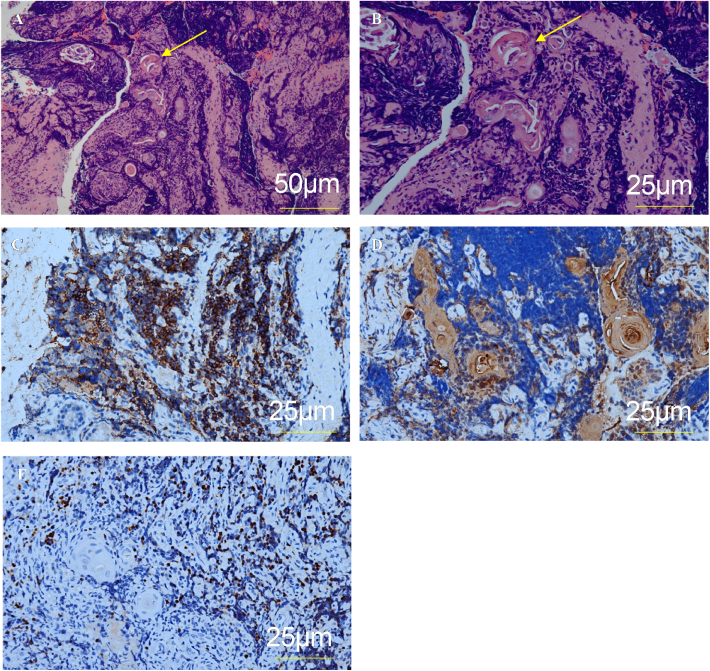
Pathohistology and immunohistochemistry. (A) Ectopic thymic tissue with Hassall corpuscles (arrow), H&E ×100. (B) Ectopic thymic tissue with Hassall corpuscles (arrow), H&E ×200. (C) CD1a+, ×200. (D) CK+ (epithelial expression), ×200. (E) TdT+, ×200.

The diagnosis of ectopic thymus was confirmed by pathohistological evaluation of the biopsy of the subglottis. Other possible etiologies (infection, autoimmune disease, benign or malignant tumor) were excluded based on the patient’s medical history, clinical presentation, laboratory and microbiological examination, and imaging studies. Postoperative pathohistological examination showed Hassall corpuscles (Fig. 4). The diagnosis of ectopic thymus was confirmed by immunohistochemistry assessment that showed positivity for CD1a, Cytokeratin (CK), and Terminal deoxynucleotidyl Transferase (TdT).

DLB was performed after removing ETT one week after the surgery. DLB showed bilateral aryepiglottic fold congestion and edema, but a patent subglottis. Mucosal healing was good, and no abnormal secretion was observed. The patient was transferred to the general ward. According to the results of sputum bacterial culture and drug sensitivity, anti-inflammatory treatment was given, followed by budesonide nebulization and anti-reflux treatment. Laryngeal obstruction was significantly relieved. No recurrence was found during two-month follow-up.

## Discussion

### Background

Cervical ectopic thymus is a rare form of congenital neck mass in children.^1^ It usually occurs in the age-group of 2–13 years children with slightly higher incidence in boys. The masses are mostly located on the left side of the neck, which may cause the change in vocal pitch, difficulty breathing, or difficulty swallowing.^2^, ^3^, ^4^ Pediatric cervical ectopic thymus generally presents as a cervical thymic cyst or ectopic thymic tissue, the latter of which was more likely found by the age of 3 years or younger.^5^ Despite the low prevalence, it is necessary to take the ectopic thymus into consideration during the differentiation diagnosis of cervical masses.

Theoretically, an ectopic thymus could occur in any other location outside the normal descend pathways, such as pharynx, trachea, posterior neck or mediastinum, or esophagus,^6^ where subglottic ectopic thymus is a rare condition. A total of 20 children with cervical thymic remnant were summarized from 1975 to 2006 at Cincinnati Children’s Hospital Medical Center, none of which were in the subglottic lesion.^7^ Up till now, only six cases of subglottic ectopic thymus have been reported separately in English literature. Basic information and characteristics of these cases were summarized in Table 1 with adding our case in this study.^5^, ^6^, ^8^, ^9^, ^10^, ^11^ Noteworthily, it is the first time to report a highly suspected subglottic ectopic thymus patient before surgical intervention.

**Table 1:** Summary of case reports of ectopic thymic tissue of the subglottic area in the English literature.

Case	Author	Age/sex	Presentation	Imaging	Diagnostic suspicions	Initial management	Definitive management	Pathohistological results
1	Martin and McAlister^5^	4 mo/F	Stridor; respiratory distress	CT; U/S; laryngoscopy	Subglottic hemangioma	Tracheotomy	Open excision	Ectopic thymus
2	Sahhar et al.^8^	10 d/M	Stridor; respiratory distress	Laryngoscopy	Subglottic cyst	Microlaryngeal excision	No further procedure	Ectopic thymic cyst
3	Pai et al.^9^	3 mo/M	Recurrent respiratory distress; stridor	CT; laryngoscopy; bone marrow trephine; CSF examination	Croup; hemangioma; Precursor T-lymphoblastic lymphoma/leukemia	IV steroids; nebulized adrenaline; chemotherapy	Open excision (modified laryngofissure approach; temporary tracheotomy)	Ectopic thymus
4	Ahmad et al.^10^	4 mo/M	Stridor; cough	Laryngoscopy	Croup	IV steroids; racemic epinephrine; microlaryngeal marsupialization	Repeat endoscopic management	Ectopic thymic cyst
5	Mohantyet al.^11^	at birth/M	Apnea	Not performed	Subglottic obstruction	Emergency tracheotomy	Autopsy	Ectopic thymus
6	Paraboschi et al.^6^	7 mo/M	Stridor	CT; MRI; U/S; laryngoscopy	Hemangioma	Propranolol	Open excision (modified laryngofissure approach)	Ectopic thymus
7	Current reported 2020	2 y/M	Stridor; cough respiratory distress	CT; MRI; U/S; laryngoscopy; bronchoscope	Ectopic thymus		Open excision (modified laryngofissure approach)	Ectopic thymus

mo, Month; d, day; y, year; F, female; M, male; CT, Computed Tomography; U/S, ultrasound; MRI, Magnetic Resonance Imaging.

### Pathophysiology

Normally, thymus tissue originates from the ventral development of bilateral third pharyngeal sacs during the sixth week of embryonic development.^12^, ^13^ Then it splits into a dorsal wing and produces the thymus.^14^, ^15^ The dorsal wing extends into the thymopharyngeal tube.^12^, ^14^, ^15^ At the eighth week, the thymopharyngeal tube moves down from the posterior sternum to the mediastinum, and fuses in the midline. This descent depends on the association with mesodermal components of the medial and posterior thymus. At the third month, the thymus is mature with cortex and medulla, and Hassall corpuscles are seen.^16^ The relative size of the thymus reaches its maximum at the age of four and begins to degenerate after puberty, but Hassall corpuscles remain.^17^

Nowadays, mainstream view of the occurrence of subglottic thymus is that could be a result of the failure descent of thymic in the embryo. If thymic precursors are pulled medially before cricoid cartilage formation at seventh week, it may be trapped within the annulus.^18^ In addition, subglottic thymus may derive from metaplasia of the subglottic pluripotent endodermal cells.^10^ However, this theory has not yet been clarified.

### Diagnosis

If a mass in the neck or anterior mediastinum is suspected of an abnormal thymus tissue, ultrasound is the first choice for the evaluation. In the published cases,^1^, ^19^ ectopic thymus presents as a clear hypoechoic lesion with punctate and linear echogenic lesions giving the characteristic “starry sky” appearance similar to the normal anterior mediastinal thymus. Ultrasonic diagnosis is based on mediastinal thymic homogeneity and similarity.^20^, ^21^

CT scan as the criterion standard for radiological diagnosis could be suggested when ultrasound results cannot provide a hint to make a definitive diagnosis.^6^ The feature of uniform low-density mass with regular edges is shown in CT scan.^20^, ^22^ A normal thymus may be barely visible on Positron Emission Tomography (PET) scans. However, it may show striking Fluorodeoxyglucose (FDG) avidity in rebound hyperplasia, causing false alarm for recurrent lymphoma.

Some authors believe that MRI scanning is the best non-invasive method to identify abnormal solid thymic problems. MRI of the normal thymus shows uniform signal intensity or slightly higher intensity compared with the muscles on T1-weighted images, and the signal intensity close to that of fat on T2-weighted images.^10^, ^23^, ^24^ Ectopic cervical thymus tissue is similar to those of mediastinal thymus. However, the cystic components of the abnormal thymus appear bright on T1-weighted images, unlike the mediastinal thymus, which appears dark on T1-weighted images.

The present preoperative examination could not accurately identify the subglottic mass as ectopic thymus tissue. Owing to these limitations and rarity, six cases of ectopic subglottic thymus previously reported in the literatures were misdiagnosed preoperatively. Indeed, a correct diagnosis can be made only by pathohistological examination of the excised specimen. Hassall corpuscles, which are restricted to the medulla and characterized by a concentric pattern of keratinization, are the most typical feature of thymus.^25^ Since thymus is mainly composed of epithelial cells and thymic lymphocytes, the immunohistochemistry shows positivity characters of these two types of cells.^25^

### Differential diagnosis

The broad differential diagnosis of pediatric neck masses typically fits into three main categories: inflammation, tumors, or structural abnormalities. There is cervical lymphadenitis, branchial arch cysts, lymphatic malformations, infantile hemangioma, squamous papilloma, parathyroid adenomas, dermoid or epidermoid cysts, carcinoids, and mucoepidermoid carcinomas in common causes.^6^, ^7^ From the perspective of the subglottic lesion, infantile hemangioma as the most common disease could be easy to confuse. Most uncomplicated cases are treated conservatively and usually without pathohistological diagnosis. Review of the previous reports revealed that three out of the six reported cases (Cases 1, 3, 6) were misdiagnosed as hemangiomas. In our case, hemangioma was successfully ruled out before the surgery without tentative treatment for hemangioma.

For differential diagnosis, imaging examination and pathohistological evaluation of the biopsy are required. Clinic radiological approach based on morphologic features is useful for evaluation of a suspected thymic mass. DLB examination should be performed to visualize the mass for proper differential diagnosis.

### Surgical management

The heterotopic subglottic thymus may cause recurrent respiratory distress and stridor. Although these symptoms can be partially alleviated by drug therapy, surgery is the gold standard for the purpose of both diagnosis and treatment, especially in cases of any symptomatic subglottic mass. A complete excision of the ectopic thymus is also important, as cases of malignant degeneration have been reported.^26^

In previous reports, several surgical approaches were used for the excision of ectopic thymus. In Case 1, under the condition of tracheotomy, a relatively unvascularized mass was found between the left superior pole of the thyroid gland and the trachea, extending backwards to the tracheoesophageal sulcus, which was completely removed. In Cases 2 and 4, thymic cysts were removed using a microlaryngoscopic surgery with a combination of cold instruments and microdebrider. No clear boundary was found between normal mucosa and the subglottic mass, and therefore marsupialization was deemed sufficient. But Case 4 repeated endoscopic surgery for the recurrence. In Cases 3 and 6, transverse neck incisions were performed to fully expose the trachea, cricoid cartilage and thyroid cartilage. Vertical midline incision was made through the cricoid cartilage to fully expose the subglottic lesions. Temporary tracheostomy or ETT inserted in the lower airways was performed for adequate exposure of the subglottic lesion in Case 3. In our case, we conducted a standard transverse neck incision, opening the cervical fascia vertically along the midline, preserving the strap muscles, and allowing a good exposure of the anterior thyroid and cricoid cartilage and the first two or three tracheal rings. The cricoid cartilage was cut vertically along the midline up to 1 cm below the vocal fold plane. An obvious mass between the perichondrium of the cricoid cartilage and the mucosa of the subglottic trachea was fully resected without tracheostomy. The intact subglottic mucosa was reconstructed to avoid the risk of postoperative stenosis.

The presence of mediastinal thymus should be confirmed before surgical resection of subglottic mass, because theoretically the removal of the thymus tissue may cause mental abnormalities, affect the development of normal immune function or induce autoimmune diseases.^20^, ^27^

### Follow-up

In the six published cases, no recurrences were found during the follow-up. In our case, a planned DLB was performed two months later, and no recurrent airway lesions were found.

## Conclusion

For children presenting with subglottic mass, the possibility of ectopic thymus should be considered in the differential diagnosis. To best of our knowledge, this is the seventh case in the English literature and the only one with a correct suspicion prior to surgery. Modified laryngofissure may be an effective approach to removing the subglottic ectopic thymic and reconstructing the intact subglottic mucosa. Pathohistological evaluation of the biopsy is the gold standard for diagnosis. The knowledge and awareness about the subglottic thymus entity enable the surgeon to make accurate diagnosis and treatment.

## Conflicts of interest

The authors declare no conflicts of interest.
